# Ecological drivers of eggshell wettability in birds

**DOI:** 10.1098/rsif.2021.0488

**Published:** 2021-10-13

**Authors:** Marie R. G. Attard, James Bowen, René Corado, Linnea S. Hall, Robert A. Dorey, Steven J. Portugal

**Affiliations:** ^1^ Department of Biological Sciences, School of Life and Environmental Sciences, Royal Holloway University of London, Egham, Surrey TW20 0EX, UK; ^2^ School of Engineering and Innovation, Open University, Milton Keynes MK7 6AA, UK; ^3^ Western Foundation of Vertebrate Zoology, Camarillo, CA 93012-8506, USA; ^4^ Department of Mechanical Engineering Sciences, University of Surrey, Guildford, Surrey GU2 7XH, UK; ^5^ The Natural History Museum, Tring, Herts HP23 6AP, UK

**Keywords:** avian, cuticle, eggshells, life-history, hydrophobic, wettability

## Abstract

Complex and at times extreme environments have pushed many bird species to develop unique eggshell surface properties to protect the embryo from external threats. Because microbes are usually transmitted into eggs by moisture, some species have evolved hydrophobic shell surfaces that resist water absorption, while also regulating heat loss and the exchange of gases. Here, we investigate the relationship between the wettability of eggshells from 441 bird species and their life-history traits. We measured the initial contact angle between sessile water droplets and the shell surface, and how far the droplet spread. Using phylogenetic comparative methods, we show that body mass, annual temperature and eggshell maculation primarily explained variance in water contact angle across eggshells. Species nesting in warm climates were more likely to exhibit highly hydrophobic eggshells than those nesting in cold climates, potentially to reduce microbial colonization. In non-passerines, immaculate eggs were found to have more hydrophobic surfaces than maculate eggshells. Droplets spread more quickly on eggshells incubated in open nests compared to domed nests, likely to decrease heat transfer from the egg. Here, we identify clear adaptations of eggshell wettability across a diverse range of nesting environments, driven by the need to retain heat and prevent microbial adhesion.

## 1. Introduction

Avian eggshells are fine tuned to the needs of the embryo and fulfil multiple adaptive functions, including crypsis, mechanical and microbial protection, gas and water exchange and providing calcium for bone growth [1,2]. For the eggshell to successfully fulfil these roles, it has to deal with environmental factors, particularly rain or incubating parents with wet plumage [1]. Bird species differ greatly in their ability to retain or repel water droplets on their eggshell surfaces [3,4]. Water-attracting (hydrophilic) eggshells may become covered by a water film, while non-wettable (hydrophobic) eggshells repel water, forming almost spherical droplets [3]. Differences in eggshell wettability reflect distinct approaches to assist embryonic development in different nesting environments, and under different parental incubation strategies. The eggshell outer surface contains thousands of microscopic pores essential for the exchange of respiratory gases, yet provide a route for pathogens to enter and infect the embryo, a key cause of disease and mortality [5]. Hydrophobic eggshells minimize water and debris coverage on the surface, and are expected to be more prevalent in those species which experience a high bacterial load in their nests, either due to nest location or parental behaviour [6]. As the presence of a water film on the shell surface reduces gas diffusion across the eggshell resulting in embryo asphyxiation [7], a strong selective pressure to repel water droplets from the shell surface, especially for eggs incubated in wet or humid areas, is expected.

**Figure 1.**
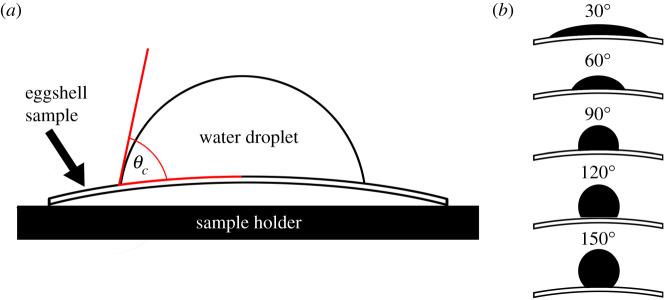
(*a*) Determination of the contact angle (*θ_c_*) between the baseline and the point of contact of a 9 µl water droplet with the surface of an eggshell sample. (*b*) When *θ_c_* is lower than 90°, the eggshell surface is termed ‘hydrophilic’, if *θ_c_* is higher than 90° and less than 150° it is termed hydrophobic. The baseline for curved eggshell surfaces uses a logarithmic function.

Surface wettability is characterized by the contact angle between a water droplet and the surface [8] and how quickly a droplet spreads across the surface [9]. A surface is hydrophilic when its static contact angle, *θ_c_* is less than 90°, hydrophobic when *θ_c_* is between 90° and 150°, and superhydrophobic when *θ_c_* is greater than 150° (figure 1) [10]. This categorization for eggshell wettability is standard practice and has been applied to numerous experimental, numerical and theoretical studies ([11], and references therein). Water-repellency and wetting phenomena on the surfaces of other natural materials provide vital clues to potential functional roles of wettability in eggshells. For example, the hydrophobic surface of bird feathers (90–120°) and superhydrophobic behaviour of lotus (*Nelumbo nucifera*) leaves (approx. 160°) enable water droplets to roll off the surface, carrying contaminants with them [12,13]. This is the ‘lotus-leaf effect’ or the ‘self-cleaning effect’ [13]. The eggs of greater flamingos (*Phoenicopterus roseus*) have evolved strongly hydrophobic shell surfaces (*θ_c_* = 113.0 ± 3.8°) [3], potentially in response to incubating their eggs in mud-platform nests that are both wet and highly humid [14]. Mound-nesting Australian bush-turkeys (*Alectura lathami*) have almost superhydrophobic eggshells (*θ_c_* = 135.3 ± 2.7°) that cause spherical water droplets to pin to the surface, trapping bacteria at the top of the droplet, preventing water from spreading and ultimately disrupting gas exchange [4]. Their highly hydrophobic eggshells have evolved in response to the microbial decomposition of the organic matter in the nest and the heat it produces [15] and is pivotal in keeping infection rates low [16]. Potential self-cleaning properties of relatively hydrophobic eggshells were not observed in common guillemots (*Uria aalge*) (approx. 91°) [3], as their eggs typically present a large amount of debris which builds up on the surface [17]. Hydrophilic eggshells have so far been reported in three bird species (brown boobies *Sula leucogaster*, domestic chickens *Gallus gallus domesticus* and helmeted guineafowl *Numida meleagris*) [3], yet only the booby nests in wet conditions, suggesting that additional factors likely influence the biological diversity of eggshell wetting properties in birds.

Eggshell wettability studies have been limited to seven species of open and ground, or mound-nesting birds [3,4], precluding any broad-scale phylogenetically informed conclusions to be drawn. Here, we encompass 441 modern bird species in 98 families to examine broad-scale patterns in shell wettability and test whether there are consistent trends with respect to life-history strategies in explaining this variation. Birds as a whole represent a highly heterogeneous group in terms of breeding locations (e.g. ground versus arboreal nesters; open versus semi-open versus dense habitats), body mass (1.9 g to 111 kg) [18] and geographic distribution [19]. Life-history traits included in our analysis covered different phases during the life of a bird, relating to embryo development, attributes of the nest and habitat, and incubation behaviour of the adult bird (table 1).

**Table 1.** Putative predictions and definitions for possible explanations for variation in eggshell wettability in birds. Source lists references for definitions and primary databases used to compile bird life-history traits. Hypotheses for variation in eggshell wettability are differentiated as either a proximate or ultimate cause. Ultimate explanations address evolutionary function (i.e. why eggshell wettability exists) and proximate explanations address the way in which the functionality is achieved (i.e. how interspecific differences in surface wettability are achieved by the eggshell). These two types of explanations complement each other and are not mutually exclusive. Hypotheses are numbered 1 to 14.

ID	predictor	cause	hypothesis	definition	source
1	body mass	proximate	as adult body mass is correlated to egg mass, eggshells of heavier birds will be less hydrophobic due to lower shell curvature.	mean body mass (g) of adult birds.	data from Dunning *et al.* [20], with updates from Wilman *et al*. [21] and Pigot *et al*. [22]. Database compiled by Sheard *et al*. [18].
2	clutch size	ultimate	evaporation from multiple eggs will create a nest atmosphere of greater humidity, so the water contact angle will be higher for species with larger clutches. More hydrophobic eggs among species with larger clutches would help reduce microbial infection.	number of eggs per brood, measured as geometric mean of the typical minimum and maximum clutch size.	databases from Jetz, Sekercioglu and Böhning-Gaese [23], Lislevand *et al*. [24] and Myhrvold *et al*. [25]. Gaps were filled in using HBW Alive [14] and other sources.
3	diet	proximate	the composition of the eggshell is influenced by diet. Plants and insects have low calcium content, so species that rely on these foods are expected to have thinner eggshells compared to species that feed on vertebrates. As microbes can more easily enter the egg of thinner eggshells, species that consume plants or insects will develop more hydrophobic eggshell surfaces to minimize microbial adhesion.	**(1) plant:** diet primarily consists of fruit, buds, seeds or plants	category based on Wilman *et al*. [21], updated from HBW Alive [14] and other sources. Database from Sheard *et al*. [18].
				**(2) insectivore:** diet primarily consists of insects	
				**(3) omnivore/carnivore:** diet is omnivorous, carnivorous or a scavenger	
4	mode of development	ultimate	longer incubation duration will promote the accumulation of microbes on the eggshell surface. Precocial species require more incubation time than altricial species, thus are expected to possess more hydrophobic eggshell surfaces.	**(1) altricial:** newly born young are relatively immobile, naked, and usually require care and feeding by the parents.	category based on Augustine *et al*. [26], Stark [27] and Stark & Ricklefs [28]. Data from HBW Alive [14] and other sources.
				**(2) precocial:** newly born young are relatively mobile, covered in feathers, and independent.	
5	egg maculation	proximate	maculated eggs are expected to be less hydrophobic if foreground and background colour have different surface roughness. Surface roughness appears to be inhomogeneous across the surface of maculated eggs [29], which may potentially increase the velocity at which water spreads across the surface.	**(1) immaculate:** no spotting or markings on eggshell surface.	category based on Portugal *et al*. [30]. Data from online museum catalogues, HBW Alive [14] and other sources.
				**(2) maculation:** maculation present on eggshell surface.	
6	nest type	ultimate	nests in cavities or burrows have a higher relative humidity than open-top nests [31] and are more insulated [32]. As the level of bacterial penetration through the shell increases with higher temperature and relative humidity [33], the shell surfaces of eggs laid in enclosed nests will be more hydrophobic than eggs laid in semi-enclosed and exposed nests.	**(1) exposed:** nest is open above and has no side walls (no nest, scrape, saucer, platform, heap).	category from this paper. Data from HBW Alive [14] and other sources.
				**(2) semi-enclosed:** nest is partially open and has side walls (cup, bowl, pendant, sphere, dome, pouch).	
				**(3) enclosed:** nest is entirely enclosed (cavity, burrow, crevice).	
7	nest location	ultimate	elevated nests have lower risk of flooding, water accumulation or exposure to dirt and animal faeces, therefore will have lower eggshell hydrophobicity compared to burrows and ground-nesting species, due to reduced risk of infections.	**(1) ground:** nest location in or on the ground.	category based on Portugal *et al*. [30]. Data from HBW Alive [14] and other sources.
				**(2) water:** floating on water.	
				**(3) elevated:** nest located in tree, bush, shrub, wall, cave roof, cliff or attached to reed.	
8	habitat	ultimate	eggs of species breeding in open habitats are more vulnerable to heat loss due to exposure to wind [34], therefore their eggshells are expected to have more hydrophobic surfaces to reduce heat loss compared to eggs of species breeding in semi-open and dense habitats.	**(1) open:** species primarily occurs in desert, grassland, open water, open moorland, low shrubs, rocky habitats, seashores and cities.	habitat scores from Tobias *et al*. [35]. Database compiled by Sheard *et al*. [18].
				**(2) semi-open:** species primarily occurs in open shrubland and bushland, scattered bushes, parkland, forest edge.	
				**(3) dense:** species primarily occurs in forest with a closed canopy, or in the lower vegetation strata of dense thickets, shrubland, mangroves or marshland.	
9	nest lining	ultimate	incorporation of nest lining will trap moisture, resulting in higher eggshell hydrophobicity.	**(1) lined:** nest lining is always or sometimes present.	category from this paper. Data from HBW Alive [14] and other sources.
				**(2) not lined:** nest lining is absent.	
10	incubating parent	ultimate	eggs are more prone to microbial penetration when the parent leaves the nest uncovered. This is more likely to occur if incubation is not shared between parents, hence these eggs are more likely to have more hydrophobic eggshells.	**(1) not shared:** contact incubation of eggs by single adult.	category from Portugal *et al*. [30]. Data from HBW Alive [14] and other sources.
				**(2) shared:** contact incubation of eggs by two adults.	
11	parental contact	ultimate	the wet incubating parent returning to the nest will increase the nest's humidity, thus eggshells of these species are expected to have higher hydrophobicity.	**(1) wet plumage:** adults return habitually to the nest with wet plumage. This included species that feed on freshwater or marine prey, or use nests built on water.	category from Portugal *et al*. [30]. Data from HBW Alive [14] and other sources.
				**(2) dry plumage:** adults did not return habitually to the nest with wet plumage.	
12	parental care	ultimate	the eggshells of species that provide biparental care are expected to be less hydrophobic, as nest humidity and temperature can be better maintained when both parents assist.	**(1) uniparental:** the brood is provisioned and/or defended by one adult	category from Portugal *et al*. [30]. Data from HBW Alive [14] and other sources.
				**(2) biparental:** the brood is provisioned and/or defended by at least two adults	
13	annual temperature	ultimate	as the level of bacterial penetration through the shell increases with higher temperature [33], eggshells of eggs incubated in warmer climates will be more hydrophobic to avoid microbial colonization.	average annual mean temperature (BIO1) of breeding/resident range.	from Sheard *et al*. [18], based on WorldClim v1 data [18].
14	annual precipitation	ultimate	eggshells incubated in environments with higher annual precipitation will be more hydrophobic, to combat temporary periods of excessive rain.	average annual mean precipitation (BIO12) of breeding/resident range.	from Sheard *et al*. [18], based on WorldClim v1 data [18].

First, (i) we examined whether eggshell wettability is influenced by shared evolutionary history between species. Secondly, (ii) we explore whether eggshell wettability is associated with life-history strategies of species, after accounting for phylogeny. We expected, under the hypothesis of eggshell surface properties being important in the evolution of bird life-history strategies, that species clustering together because of similar life-history traits would share similar degrees of eggshell surface wettability. We predicted that species breeding in hotter and wetter environments will tend to have more hydrophobic eggshell surfaces to protect their eggs against microbial infection [3]. Similarly, eggs in exposed, unsheltered nests are more vulnerable to heat loss due to exposure to wind [34], therefore were expected also to have more hydrophobic surfaces.

## 2. Material and methods

### 2.1. Eggshell sampling

Eggshells from 441 species (1508 eggs) were sampled from the Western Foundation of Vertebrate Zoology at Camarillo (USA) and the Class II (i.e. data poor) egg collection at Natural History Museum at Tring (UK). Combined, these museums hold the largest research collection of blown bird eggs worldwide [36,37]. This dataset incorporates species across a range of body masses, from hummingbirds to ratites. We excluded eggs that were too small to rest a water droplet on (minimum egg length used was 1.5 cm).

We previously determined that old eggs can be used [38] as detailed in the electronic supplementary material. Only eggs from early stages of incubation were selected for this study, based on the size of the blow hole [39] or information on the incubation stage stored with the clutch. For sample collection, whole emptied eggshells were either cut in half along with the longitudinal-axis (pole-to-pole) or a square (approx. 1.5 cm × 1.5 cm) was cut from the equatorial region of each shell using a micro-tool rotary saw with a diamond-coated cutting wheel (Dremel 4000, Bosch Leinfelden, GER). Fragments from up to 24 eggs were sampled per species. Eggshells were gently cleaned with a cotton bud dipped in distilled water, then dried for at least 24 h at room temperature.

### 2.2. Wettability measurements

Eggshell wettability was quantified using contact angle goniometry. Contact angles were measured by the sessile drop method, using a Krüss (Germany) Drop Shape Analysis System (EasyDrop Standard) and Advance software (version 1.8-01). A drop of liquid placed onto a solid surface shows a characteristic wetting angle (contact angle *θ_c_*) between the baseline of the drop and the tangent line at the liquid–solid intersection and is compliant with curved surfaces including eggshells [40] (figure 1). To create the baseline, the Advance software applied a trigonometric function to describe the curved eggshell surface. The wettability of a solid is defined as: hydrophilic = *θ_c_* < 90° hydrophobic = 90° < *θ_c_* < 150°; superhydrophobic = *θ_c_* > 150° [10]. We measured the contact angle behaviour of a 9 μl droplet of deionized water (dispense rate: 3 µl s^−1^) on the surface of the eggshell equator. This region was chosen as it has less variation in shell curvature [41] and thickness [42] than either end of the egg. This droplet volume was chosen in a previous study [3] to give droplet diameters (approx. 2.5 mm) several orders of magnitude greater than the length scale of cuticle roughness/nanospheres [43]. Once the droplet formed at the syringe tip, the stage holding the shell fragment was gently moved upwards until the droplet base touched the eggshell surface. The fragment was then lowered away from the syringe tip, taking the droplet with it. Images were recorded over the next 25 s at 2 Hz (see video in electronic supplementary material). We used the Young–Laplace fitting model to determine the static contact angle, which applies a surface curvature to the droplet [10]. At each time point, contact angle at the left and right side of the droplet was averaged to obtain *θ_c_*. On occasion, the Advance software was momentarily unable to locate the water droplet; therefore, *θ_c_* was averaged across the first 2 s of contact between the droplet and shell fragment. This measurement was repeated at two locations on the same fragment for a subset of samples to assess the repeatability of measurements from the fragment level. We first calculated within-fragment repeatability using the *rpt.aov* function in package ‘rptR’ for Gaussian data distribution using 1000 permutations [44]. Paired *t*-tests were used to compare wettability measures between paired locations on the same fragment.

According to Wenzel [45], a liquid droplet resting on a solid surface will have a specific energy content that will be different for the wetted area under the drop, than the dry area around it. If the wetted area has lower specific energy, the water droplet will spread, thereby increasing the surface area under the droplet and the surface area of the liquid–air interface. Thus, spreading of the droplet immediately after contact forms a wetting characteristic of a solid. A low viscosity liquid such as water is expected to achieve equilibrium relatively quickly, with additional spreading indicating time-dependent phenomena such as the infiltration of liquid into sub-surface pores and through surface asperities, both of which are expected for the heterogeneous topographies presented by these eggshells. The decrease in *θ_c_* over time can also be explained by evaporation [45], and the initial droplet shape determines the evaporation time [46]. Hence, the difference in contact angle between 0 s and 20 s was used to quantify the wetting of the surface, herein called spreadability (Δ*θ*_C_), where *Δθ_c_* = *θ_c_*_(*t* = 0)_ *θ_c_*_(*t* = 20s)_. Positive values for Δ*θ_c_* indicate that *θ_c_* decreases. For example, if *θ_c_*_(*t* = 0)_ was 100° and Δ*θ_c_* is 30°, after 20 sec *θ_c_*_(*t* = 20s)_ is 70°.

### 2.3. Life-history and ecological data

We collected literature data on 21 key life-history traits (table 1; electronic supplementary material, table S1) for different ultimate (ecological) and proximate (mechanistic) causes of eggshell wettability across modern birds. Ultimate explanations are concerned with why eggshell wettability exists (e.g. parental nesting and foraging behaviours can influence the humidity and microbial load in the nest [30], which enhances fitness benefits of the trait), and proximate explanations are concerned with how it works (e.g. is eggshell wettability caused by differences in roughness or structural features on the shell surface, or curvature of the egg). These two types of explanation are complementary, and we must consider both to completely understand evolutionary explanations of trait divergence [47].

Of the life-history traits considered, 14 of these were included in the final analysis following removal of parameters due to multi-collinearity based on paired correlations (electronic supplementary material, figure S1) and variance inflation factor (electronic supplementary material, tables S2–S5) (see electronic supplementary material for details). Table 1 lists the hypotheses, causes and definitions of the final predictors. The number of species in each category is listed in the electronic supplementary material, table S6. All data and R scripts are in the Figshare repository (doi:10.6084/m9.figshare.14685744) and all sources for life-history data are in the electronic supplementary material and Figshare repository.

### 2.4. Statistical analysis

Analyses and plots were processed using R v3.6.1 [48]. Cook's distance was applied to specimen *θ_c_* values to identify outliers and/or influential values, which were subsequently removed from analysis. Phylogenetic signal is the extent that trait covariation reflects their shared evolutionary history, as approximated by Brownian motion [49]. Pagel's lambda (*λ*) was used to determine the extent of phylogenetic signal for each response variable using the *phylosig* function in the package ‘phytools’ [50]. At *λ* = 0 the trait of interest may vary randomly across a phylogeny while at *λ* = 1, closely related species tend to exhibit more similarity in trait expression [51].

Adult body mass accounts for much of the variation in life-history patterns among birds [52]. To control for the effects of body mass, separate analyses were performed whereby wettability measures were regressed against body mass, then the residuals from the regression were compared across life-history traits. To achieve this, we ran two series of phylogenetic analyses for *θ_c_* and Δ*θ_c_*. Our first set of phylogenetic analyses includes adult body mass as a predictor, and *θ_c_* or Δ*θ_c_* as the response. We then ran separate phylogenetic generalized least-squares (PGLS) regressions for *θ_c_* and Δ*θ_c_* against log(body mass) (slope = −4.32 ± 0.73 s.e.; intercept = 111.95 ± 1.50 s.e.; *λ* = < 0.001 for *θ_c_* and slope = 1.59 ± 1.10 s.e.; intercept = 15.19 ± 4.91 s.e.; *λ* = 0.58 for Δ*θ_c_*; electronic supplementary material, figure S2) to extract their residuals. These residuals, herein called relative contact angle (R*θ_c_*) and relative spreadability (RΔ*θ_c_*), represent mass-corrected values and were used as response variables for the second series of phylogenetic analyses whereby adult body mass was removed as a predictor. Phylogenetic comparative analyses using the residual response variables enabled us to ascertain how well one or more predictors influences the eggshell wettability properties of a species than expected for its adult body mass. All numeric predictors, except annual temperature, were log_10_-transformed prior to phylogenetic analysis to reduce skewness, as determined through visual inspection of histograms and using the *skewness* function in package ‘moments’ [48].

**Figure 2.**
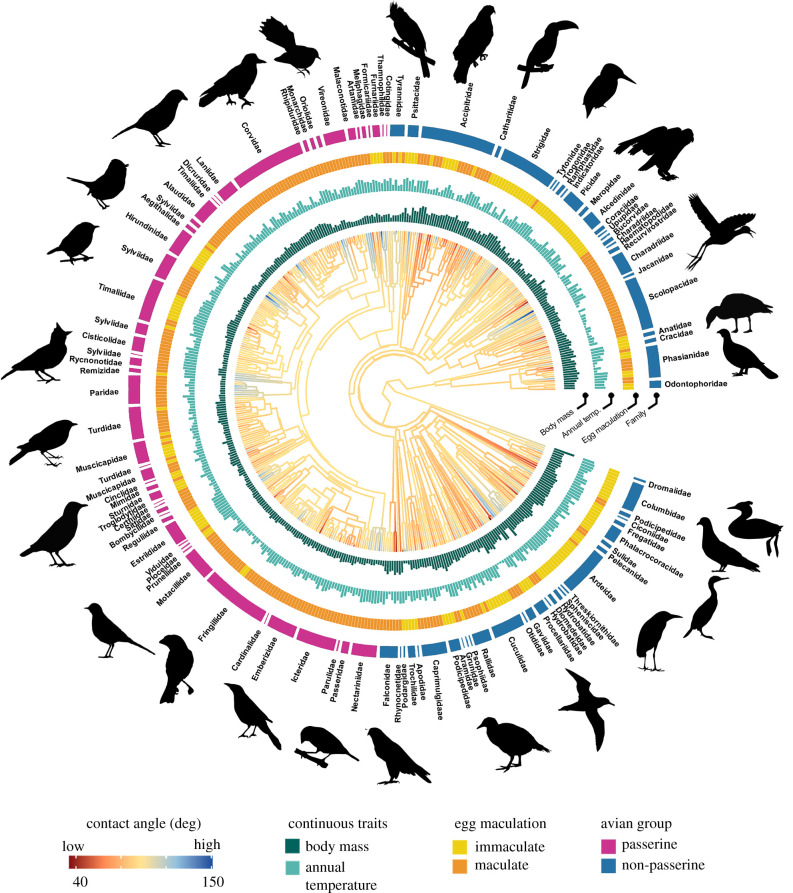
Phylogenetic tree of contact angle of eggshells for 441 bird species. Bar plots and rings around the phylogeny represent significant predictors of water contact angle (*θ_c_*) in conditionally averaged models. Branch colours show the diversification in *θ_c_* across the phylogeny and branch lengths show ancestral trait estimates. Passerines (pink) and non-passerines (blue) were analysed both together and separately. The bar plots and other rings around the phylogeny represent significant predictors of *θ_c_* in conditionally averaged models. Silhouette illustrations came from PhyloPic (http://phylopic.org), contributed by various authors under public domain licence (see electronic supplementary material).

**Figure 3.**
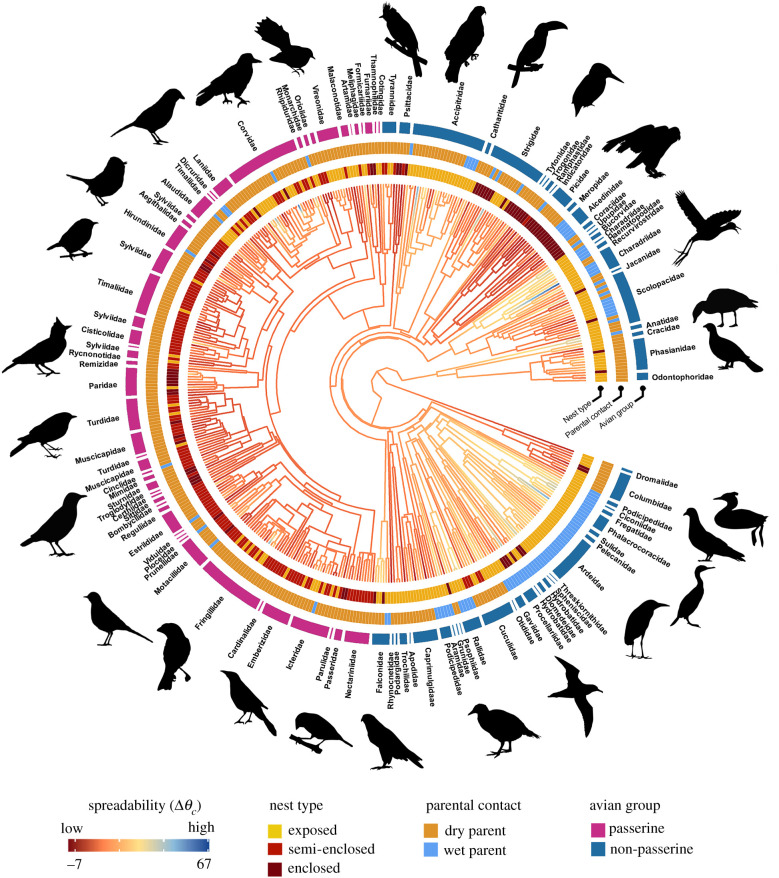
Phylogenetic tree of water droplet spreadability (Δ*θ_c_*) on eggshell surfaces across 441 bird species. Branch colours show diversity in Δ*θ_c_* and branch lengths show ancestral trait estimates. Passerines (pink) and non-passerines (blue) were analysed both together and separately. Other rings around the phylogeny represent the most influential predictors of Δ*θ_c_*. Silhouette illustrations came from PhyloPic (http://phylopic.org), contributed by various authors under public domain licence (see electronic supplementary material).

The association between eggshell wettability properties and life-history traits was assessed using PGLS regression to correct for phylogenetic non-independence of the data [53]. We generated a set of 10 000 trees constructed online (http://www.birdtree.org) using the primary backbone tree of Hackett *et al*. [47] for all species in our dataset and summarized these into a single consensual tree (figures 2 and 3). PGLS models were fitted in the package ‘phylolm’ using the *phylolm* function [54]. We first ran a full model containing all traits as predictor variables, then fitted all possible model combinations with a maximum of five predictors using the *pdredge* function in package ‘MuMIn’ [55]. This included a null model comprising only the intercept. We retained all models with ΔAICc < 2 from the model with the lowest AICc [56]. Conditional model averaging was then used to quantify the importance of each trait present in the retained models [57]. We first analysed all species as a whole, then examined whether any traits we found to be significant for all birds varied between non-passerines (*n* = 209) and passerines (*n* = 232) by analysing these taxonomic subdivisions separately. Mode of development and parental care was excluded as predictors from phylogenetic analyses within passerines as all species are altricial and most show biparental care (electronic supplementary material, table S6).

To confirm that our main results are robust to the error generated by uncertainties due to within-species variation in eggshell wettability, we ran generalized linear mixed models with Markov chain Monte Carlo (MCMCglmm) estimation methods implemented in the ‘MCMCglmm’ package [58]. This approach, based on Bayesian mixed effect models, enabled us to incorporate within-species variation in *θ_c_* and Δ*θ_c_* by fitting individual-level data and, at the same time, to control for non-independences in species traits due to common ancestry [59]. We used a single consensual tree, and applied 130 000 iterations, 100 thinning intervals and 30 000 burnin. Phylogeny was included in the models as a random effect, together with species level, to control for phylogenetic non-independence and non-independence due to factors unrelated to phylogeny [59]. In the main model, individual-level wettability values were set as a dependent variable and the life-history traits were included as fixed effects.

## 3. Results

### 3.1. Repeatability within eggshell fragments

We found that both *θ_c_* (*r* = 0.44 + 0.03, 95% CI: 0.37, 0.50, p.aov < 0.001, p.permut = 0.001 *n* = 1196 droplets, 598 fragments) and Δ*θ_c_* (*r* = 0.44 + 0.03, 95% CI: 0.37, 0.50, p.aov < 00.001, p.permut = 0.001, *n* = 1156 droplets, 578 fragments) were significantly repeatable between locations on the same shell fragment. Paired *t*-tests confirmed no significant difference in *θ_c_* (*t* = −0.66, d.f. = 619, *p* = 0.51) or Δ*θ_c_* (*t* = 1.32, d.f. = 577, *p* = 0.19) between paired locations on the same fragment. All values from the same fragment were thus averaged to a single specimen value. Mean *θ_c_* and Δ *θ_c_* were calculated for each species using single specimen values.

### 3.2. Inter-species variation in water contact angle

The *θ_c_* and Δ*θ_c_* of water on eggshell surfaces varied significantly across bird species (electronic supplementary material, figure S3). Of the 441 species analysed, 89% had hydrophobic eggshells and 11% had hydrophilic eggshells, based on prior definitions (see Methods). The spreading rates of sessile water droplets on most eggs was highest immediately after the droplet formed on the surface, then gradually reduced over time (see video in electronic supplementary material). In most eggs, we observed that droplet shape reached a steady state at approximately 15–20 s after initial contact.

### 3.3. Phylogenetic signal

Pagel's *λ* for *θ_c_* (*λ* = 0.08) and R*θ_c_* (*λ* = 0.07) were significantly different from 1 (*p* < 0.001); however, only R*θ_c_* was significantly different from 0 (*p* = 0.25 for *θ_c_* and *p* < 0.01 for R*θ_c_*), indicating that *θ_c_* has evolved independently of phylogeny, but to a slightly lesser extent after accounting for allometry. Thus, close relatives are not more similar on average than distant relatives for this trait (table 2). Pagel's *λ* was intermediate and significantly different from 0 and 1 for Δ*θ_c_* (*λ* = 0.68, *p <* 0.001 for null hypothesis that *λ* = 0 or *λ* = 1) and RΔ*θ_c_* (*λ* = 0.64, *p* < 0.001 for null hypothesis that *λ* = 0 or *λ* = 1), indicating that trait variation is associated with phylogeny and evolutionary processes other than pure Brownian motion (where a trait value changes randomly in both direction and distance over time) [60].

**Table 2.** Estimates of phylogenetic signal in contact angle (*θ*c) and spreadability (Δ*θ*c) in all birds. Phylogenetic signal was analysed separately for residual response variables. The *p*-value tests the null hypothesis for both no phylogenetic signal (*λ* = 0) and a Brownian motion model (*λ* = 1) of evolution.

response variable	Pagel's *λ*	log likelihood	log likelihood for *λ* = 0	log likelihood for *λ* = 1	*p* for *λ* = 0	*p* for *λ* = 1
contact angle	0.08	−1710.13	−1710.79	−1894.91	0.25	<0.001
residual contact angle	0.07	−1691.50	−1695.46	−1889.88	<0.01	<0.001
spreadability	0.68	−1611.08	−1668.05	−1762.51	<0.001	<0.001
residual spreadability	0.64	−1611.04	−1654.79	−1762.69	<0.001	<0.001

### 3.4. Phylogenetic comparative analysis across birds

#### 3.4.1. Water droplet contact angle

**Figure 4.**
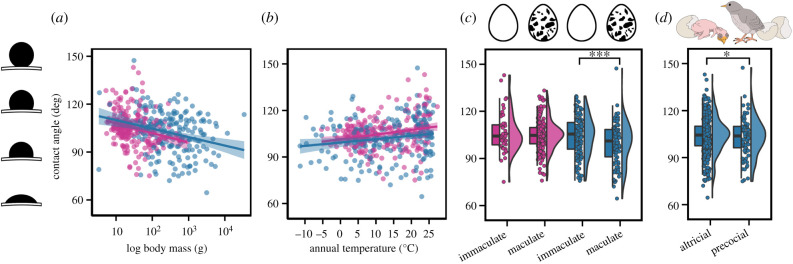
Contact angle (*θ_c_*) of a water droplet to eggshell surface plotted as a function of (*a*) adult body mass, (*b*) annual temperature, (*c*) eggshell maculation and (*d*) mode of development. Passerines (pink) and non-passerines (blue) were analysed separately, and each data point represents the mean *θ_c_* for a given species. Linear regressions for each avian group are applied in (*a*) and (*b*). Within each avian group and across all birds, *θ_c_* significantly decreased with larger body mass and increased with higher annual temperature. In the hybrid box plots (*c,d*), species *θ_c_* are shown as filled circles, vertical line indicates the median, box shows the interquartile range (IQR) and the whiskers are 1.5 × IQR and their distribution are shown as histograms. Significant differences between categorical variables based on conditionally averaged models are given in asterisks with ***p < 0.001, **p < 0.01and *p < 0.05.

We found strong support for the hypotheses that variation in *θ_c_* in modern birds is influenced by body mass, annual temperature, eggshell maculation, mode of development and nest type (figure 4; electronic supplementary material, table S7–S11). Across all phylogenetic models fitted to the global dataset, *θ_c_* was negatively associated with adult body mass (*pgls* and *MCMCglmm*, *p* < 0.001; electronic supplementary material, table S7–S9). As adult body mass is strongly and positively correlated to eggshell thickness (*R^2^* = 0.96) and fresh egg mass (*R^2^* = 0.97) (electronic supplementary material, figure S1), this would suggest that smaller, thinner eggshells are more likely to have a higher *θ_c_* than larger, thicker eggshells. The annual temperature ranged from −11°C (snowy owls, *Bubo scandiaca*) to 27°C (common gonoleks, *Laniarius barbarus*) among species included in this study. Overall, the eggs of species in warmer climates had a higher *θ_c_* and R*θ_c_* than species breeding in cooler climates (*pgls*, *p* < 0.001; figure 3*b*). In general, species with immaculate (i.e. non-patterned) eggs had a higher *θ_c_* and R*θ_c_* (*pgls*, *p* = 0.01 for *θ_c_* and R*θ_c_*) than maculated (i.e. patterned) eggs. Contact angle was significantly higher in precocial species than altricial species both before and after accounting for body mass (*pgls*, *p* = 0.01). MCMCglmm models also indicate that species with semi-enclosed nests lay eggs with higher *θ_c_* than species with exposed nests (*p* = 0.03; electronic supplementary material, table S9).

#### 3.4.2. Water droplet spreadability

**Figure 5.**
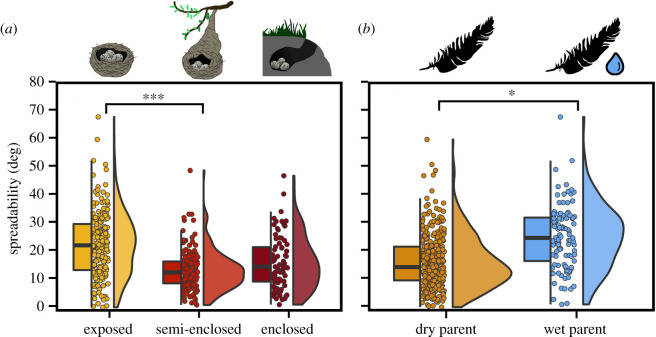
Water spreadability (Δ*θ_c_*) plotted across all species as a function of (*a*) nest type, and across non-passerines as a function of (*b*) whether the incubating bird returned to the nest with wet or dry plumage. In hybrid box plots, species mean contact angle are shown as filled circles, vertical line indicates the median, box shows the IQR and the whiskers are 1.5 × IQR and their distribution is shown as histograms. Significant differences between categorical variables based on conditionally averaged models are given in asterisks with ***p < 0.001, **p < 0.01 and *p < 0.05.

Variation in Δ*θ_c_* across bird species was primarily associated with nest type and whether the incubating parent returns to the nest with wet or dry plumage (figure 5; electronic supplementary material, table S12–S17). The significantly low Δ*θ_c_* of droplets in semi-enclosed nests compared to exposed nests (*pgls*, *p* < 0.001; MCMCglmm, *p* = 0.001; figure 5*a*) persisted after accounting for differences in adult body mass (*pgls*, *p* < 0.001; electronic supplementary material, table S13). MCMCglmm results further showed that eggs in enclosed nests have significantly low Δ*θ_c_* compared to exposed nests (*p* = 0.04, electronic supplementary material, table S14). Droplets also spread more rapidly on eggshells of species where parents typically return to the nest with wet plumage (*pgls*, *p* = 0.02 for Δ*θ_c_* and *p* = 0.03 for RΔ*θ_c_*). Other features of the nest, such as the presence of nest lining or nest location (water, ground or elevated) had no significant influence on *θ_c_* or Δ*θ_c_* in all phylogenetic analyses (electronic supplementary material, table S7–S16).

### 3.5. Phylogenetic comparative analysis within passerines and non-passerines

#### 3.5.1. Water droplet contact angle

When passerines and non-passerines were analysed separately (electronic supplementary material, table S17–S36), both groups experienced higher *θ_c_* with decreased adult body mass (*pgls, p* < 0.01 and MCMCglmm, *p* = 0.01 for passerines; *pgls* and MCMCglmm, *p* < 0.001 for non-passerines) and increased average annual temperature of their breeding range (*pgls, p* = 0.01 for passerines and non-passerines). Contact angle among non-passerines was also significantly higher for species with immaculate eggs (*pgls*, *p* = 0.01 figure 4*c*), precocial young (*pgls*, *p* < 0.05) or located in open habitats compared to semi-open (*pgls*, *p* = 0.01) (electronic supplementary material, table S29). MCMCglmm models also indicate that non-passerines with semi-enclosed nests lay eggs with higher *θ_c_* than species with exposed nests (*p* = 0.03; electronic supplementary material, table S35).

#### 3.5.2. Water droplet spreadability

Annual temperature and nest type influenced Δ*θ_c_* in passerines, both before and after accounting for differences in body mass (electronic supplementary material, table S21–S24). Among passerines, Δ*θ_c_* was significantly lower in eggs of species that use semi-enclosed nests than both other nest types (*pgls*, *p* < 0.001 and MCMCglmm, *p* = 0.02 for exposed nests, and *pgls*, *p* = 0.02 for enclosed nests), even after accounting for differences in body mass (*pgls*, *p* < 0.001 for exposed nests, and *pgls*, *p* = 0.02 for enclosed nests). Increased annual temperature resulted in higher Δ*θ_c_* and RΔ*θ_c_* among passerine eggs (*pgls, p* = 0.02 for Δ*θ_c_* and *p* = 0.01 for RΔ*θ_c_*) (electronic supplementary material, table S21 and S23).

Among non-passerines, species that return to the nest with wet plumage had significantly higher Δ*θ_c_* of droplets on their eggs compared to species that return to the nest with dry plumage (*pgls*, *p* = 0.04; electronic supplementary material, table S31), but was not significant after accounting for adult body mass (*pgls*, *p* = 0.09; electronic supplementary material, table S33).

## 4. Discussion

Variation in eggshell wettability across birds was influenced by phylogenetic relatedness and differences in life history. We found support for the hypotheses that species breeding in warmer climates, with precocial young or with immaculate shells had less wettable surfaces. Birds with lighter body masses also produced eggs that exhibited greater *θ_c_*. Moreover, the influence of body mass and annual temperature on *θ_c_* persisted when non-passerines and passerines were analysed separately. Eggshell pigmentation was a significant predictor of *θ_c_* for all birds, but when separate, not for passerines. Among non-passerines, eggs of species with precocial young had higher *θ_c_* than altrical young after accounting for differences in adult body mass. Habitat type was another significant predictor of *θ_c_* in non-passerines, with eggs of species in open habitats having more hydrophobic properties than eggs of species in semi-open habitats. By contrast, variation in Δ*θ_c_* on eggs across bird species was primarily associated with nest type and whether the incubating parent habitually returns to the nest with wet or dry plumage. Species with wet plumage are those that feed on marine or freshwater prey or use nests built on water.

### 4.1. Anti-microbial defence

Functionally, the formation of more spherical water droplets due to greater water repellence ensures that minimal water is in direct contact with the shell surface [4], thereby eliminating microbial film formation on the shell surface itself or in/over pores. Hydrophobic eggshells may be particularly advantageous for species breeding in warm and wet environments where microbes are more prolific [61]. Among the most hydrophobic eggs were common gonoleks (*Laniarius barbarus*) (*θ_c_* = 122.39, Δ*θ_c_* = 3.37) and African paradise-flycatchers (*Terpsiphone viridius*) (*θ_c_* = 122.56, Δ*θ_c_* = 3.48), both of which breed in wooded savannas during the wet season [14], thus anti-microbial surface properties would be advantageous. Budgerigars (*Melopsittacus undulatus*) breed in open habitats (open forests and plains, savanna and deserts) any time of year after substantial rain when there is likely to be a lot of water present in the environment and likewise have highly hydrophobic eggshells (*θ_c_* = 129.69, Δ*θ_c_* = 11.32). Most grebes and coots lay eggs in nests floating on water, and it has been noted that these eggs come in contact with, and often partially submerged in water [62]. American coots (*Fulica americana*) had hydrophobic eggshells (*θ_c_* = 98.58, Δ*θ_c_ =* 30.08) and three of the four grebe species included in our study had hydrophobic eggshells (*θ_c_* = 107.73–113.93, Δ*θ_c_* = 10.14–16.00) based on *θ_c_*, while eggshells of the black-necked grebe (*Podiceps nigricollis*) were nearly hydrophobic (*θ_c_* = 89.34, Δ*θ_c_* = 22.56). Overall, these findings are consistent with the idea that species incubating their eggs in warm, humid conditions are more likely to display hydrophobic surfaces to assist in reducing the risk of microbial infection [4]. Increased incubation duration also promotes the accumulation of microbes on the eggshell surface; therefore more hydrophobic eggshells reported among precocial species will be beneficial given their longer incubation time compared to altricial species.

### 4.2. Eggshell maculation and roughness

Many studies have shown direct and indirect impacts of pigmentation on eggshell properties, including shell ultrastructure [63,64], shell thickness distribution [2,65] and eggshell permeability [66]. We propose that the degree of maculation (spotting) on an eggshell has a direct impact on its wettability in non-passerines. Rough surfaces produce matte eggshells [67] and require a greater decrease in energy than smooth surfaces to induce spreading. Eggshells with higher surface roughness also spread droplets more rapidly than a smooth eggshell [68] and have a lower droplet height [69]. Surface roughness has not previously been quantified between background pigmentation (base colour) and foreground (top colour) pigmentation of maculated bird eggs; however, heterogeneity in surface structure has been reported in maculated eggshells. Mróz *et al*. [64] observed that the pigment-covered areas of turkey eggshells have a different surface structure than the base colour of maculated eggs, while the base colour of maculated eggs was similar in surface structure to immaculate eggs. This suggests surface roughness is inhomogeneous across the surface of patterned eggs, which may increase the water spreading velocity across the surface [29]. Velocity would also depend on the type of pattern. For example, if the pattern was isolated (e.g. some eggs have a ring of foreground pigmentation concentrated towards the blunt end), there could be a local advancement of a water drop, but the velocity would still largely depend on the base colour. If the pattern was evenly distributed throughout the eggshell, the spreading velocity would depend on both background and foreground pigmentation. As *θ_c_* was higher in immaculate eggshells than maculate eggshells, we would predict that immaculate eggshells are smoother than maculated eggs, and foreground pigmentation has greater surface roughness than the background pigmentation of maculated eggs. Both the topographical and chemical properties of the eggshell surface are important for wetting and adhesion behaviour, requiring further attention to resolve the key mechanism behind these phenomena.

### 4.3. Contact angle associated with eggshell curvature

The strong negative association between *θ_c_* on the eggshell and adult body mass indicates that smaller eggs tend to be more hydrophobic. The shape of water droplets varies between flat and curved surfaces [70], which may explain the disparity in *θ*_*c*_ between different sized eggs. A drop hitting a flat surface retains a circular symmetry throughout the impact process [71]. Flat surfaces with a smaller *θ_c_* have a higher droplet spreading factor (ratio of length of two-dimensional wetting arc and initial droplet diameter [72]) and reach equilibrium sooner than flat surfaces with a large *θ_c_* [9]. The rate of droplet spreading becomes slower with decreasing curvature ratio (the ratio of the initial droplet diameter to the surface diameter) [73]. With droplet volume fixed, the *θ_c_* for a spherical surface with a smaller diameter (high curvature ratio) is higher than with a larger diameter (low curvature ratio) [74]. This implies that a smaller *θ_c_* is induced in larger eggs of a given egg shape. However, complete wetting of the surface is expected when the droplet-to-sphere ratio is close to unity, so a higher curvature ratio in species with very small eggs, like red-breasted swallows (*Hirundo semirufa*; *θ_c_* = 143°), does not necessarily protect the shell from water coverage. Staying wet means being colder as heat is withdrawn during evaporation of surface water [75], which must be considered disadvantageous for smaller eggs given their larger surface-to-volume ratio, so consequently will lose more heat when drying.

### 4.4. Nest type and heat transfer

We suggest that the high Δ*θ_c_* on the shell surfaces of species that incubate their eggs in exposed nests (cups, platforms, scrapes and depressions) may reflect their need to decrease heat transfer from the egg, yet further research is required to support our supposition. Wind has greater influence on heat loss than conduction and evaporation [76]. Reducing air movement over bird's eggs moderates their convective heat loss, which is achievable using sheltered microsites such as cavities and domed nests [77]. Water vapour condenses on a surface either by ‘dropwise’ or ‘filmwise’ condensation [78], which influences heat transfer. Filmwise condensation forms a film of vapour across a wettable surface, increasing in thickness as it flows downwards, as additional vapour is picked up along the way. Dropwise condensation occurs when droplets form an acute angle to a non-wettable surface and will gather all the static droplets as it flows downwards, leaving behind a bare trail. The bare surface offers very little resistance to heat transfer and can result in high heat fluxes. Consequently, dropwise condensation on the eggshell surface (high *θ_c_* and low Δ*θ_c_*) is likely to produce a heat transfer coefficient an order of magnitude higher than the filmwise condensation (low *θ_c_* or high Δ*θ_c_*) [79]. Thus, heat loss from eggs can be reduced in unsheltered microsites by increasing the spreadability of droplets on their shell surface.

The influence of eggshell surface properties on evaporation and subsequent heat loss from the egg are complex, with multiple contributing factors. A rough shell surface is more likely to disturb the air movements across the shell surface and reduce the rate of droplet evaporation and heat transfer from the egg compared to a smooth surface, as demonstrated in other microstructures [80]. Natural evaporation of sessile water droplets on a hydrophilic surface is quicker than on a hydrophobic surface [81], thereby cooling the egg more rapidly. This is because the contact area between the water droplet and the eggshell surface is much higher for hydrophilic surfaces, and thus, these surfaces will experience a higher heat transfer rate and quicker evaporation. However, as mentioned earlier, a bare surface will be more vulnerable to high heat fluxes. Water droplets also evaporate more rapidly on a surface with high contact angle hysteresis, which would be advantageous in ‘dirty’ nests by quickly removing water required for microbial growth. The drying attributes of eggshells are further complicated by its porous surface, which likely comprise ‘liquid’ (water-filled) pores and ‘gas’ (empty) pores.

In the case of exposed nests, the formation of discrete water drops or water films on eggs can have multiple conflicting effects. The focusing properties of rounder water droplets on biological surfaces could become an issue for eggs in unsheltered nests due to their direct exposure to sunlight including UV [82]. Water droplets left on shell surfaces in direct sunlight may act to focus and increase the light intensity multiple times more directly beneath individual droplets [83], which then can penetrate the shell and damage the embryo [82]. Species in exposed nests (platforms, cups, scrapes and depressions) may benefit from hydrophilic eggshell properties by reducing light intensity on its surface if they become wet. However, water droplets also tend to dry faster in exposed locations, which could lead to evaporative cooling of the eggshells, while simultaneously reducing the potential ‘focusing’ effects of the sun. We speculate that this focusing effect is unlikely to be a natural selective driver in situations where eggshells do not stay wet for long. The importance of protecting embryos from UV-light transmission is likely to increase with decreasing shell thickness. Because eggshell thickness at a pigment spot is significantly thinner than the adjacent unpigmented shell region [2], we would anticipate that maculated eggs or those with thinner shells than expected for their size are more likely to develop hydrophilic surfaces to minimize embryo exposure to heat and harmful UV.

### 4.5. Wettability of other natural surfaces

The large range of eggshell wettability among the eggs of bird species likely has strong effects on gas exchange, embryo growth and risk of infections. Wettability variation also correlates with challenging environments, such as those where the eggs frequently become wet or are exposed to very dry air. The selective pressures promoting the evolution of hydrophobicity on the outmost layer of other organisms are highly varied, but like eggshells, are usually based on interactions with wet or dirty environments. Hydrophobic leaves with low water droplet retention are more commonly found in plants from arid/semi-arid regions, or open habitats where water availability is limited by allowing water to fall from leaves to the soil [84,85]. Tenebrionid beetles (*Stenocara* sp.) use patterned hydrophobic and hydrophilic surface structures on their backs to capture early-morning fog that rolls down into their mouth so they can survive arid environments [86]. Many insects living in or near aquatic environments, such as dragonflies [87] and termites [88], possess hydrophobic wings to maintain adequate mobility when wet. By contrast, the hydrophilic leaf-shaped wings of black cicadas (*Gudanga* sp.) retain condensed moisture to serve as camouflage in dense foliage and reflect their sedentary lifestyle, with limited flying [89]. Dense hydrophobic fur insulates semi-aquatic mammals without restricting terrestrial movement and increases floating capacity, but incurs a high energy demand for maintenance and limits diving depth [90]. Thus, it seems that the demands placed upon the eggshell surface shares some similarities with general patterns exhibited in other taxa. For future research, repeated measures in eggshell wettability over the incubation period across multiple ecologically diverse bird species would be beneficial to understanding (i) to what extent does eggshell surface properties change over the incubation period and (ii) how the embryo is impacted by transitional physiological and mechanical capabilities of the shell. Global variation in multiple avian eggshell surface properties (e.g. wettability, eggshell colour and luminescence [91]) is strongly predicted by the temperature of a species' breeding range. Climate-driving changes in breeding performance across birds are likely to be influenced by these traits, and species that persist in future climates may be able to do so in part owing to the adaptability of their eggshells to environmental challenges.

## Data accessibility

Data and R scripts are available from the Figshare Repository (doi:10.6084/m9.figshare.14685744). All results for phylogenetic comparative analysis are provided in the electronic supplementary material [92].
